# Large-scale investigation confirms TRPM3 ion channel dysfunction in Myalgic Encephalomyelitis/Chronic Fatigue Syndrome

**DOI:** 10.3389/fmed.2025.1703924

**Published:** 2026-01-08

**Authors:** Etianne Martini Sasso, Teagan S. Er, Natalie Eaton-Fitch, Livia Hool, Katsuhiko Muraki, Sonya Marshall-Gradisnik

**Affiliations:** 1 The National Centre for Neuroimmunology and Emerging Diseases, Griffith University, Gold Coast, QLD, Australia; 2 Consortium Health International for Myalgic Encephalomyelitis, National Centre for Neuroimmunology and Emerging Diseases, Griffith University, Gold Coast, QLD, Australia; 3 School of Human Sciences (Physiology), The University of Western Australia, Perth, WA, Australia; 4 Victor Chang Cardiac Research Institute, Darlinghurst, NSW, Australia; 5 Laboratory of Cellular Pharmacology, School of Pharmacy, Aichi-Gakuin University, Nagoya, Japan

**Keywords:** calcium, ME/CFS, Myalgic Encephalomyelitis/Chronic Fatigue Syndrome, transient receptor potential melastatin 3, TRP ion channels, TRPM3

## Abstract

**Introduction:**

Myalgic Encephalomyelitis/Chronic Fatigue Syndrome (ME/CFS) is a chronic disease hallmarked by multiple systemic symptoms, such as neurocognitive, respiratory, immunological, gastrointestinal, and cardiovascular impairment, which worsen following physical and mental exertion. ME/CFS is characterized by an elusive pathomechanism, profound impact on quality of life, and an absence of diagnostic tests or evidence-based treatments. Transient Receptor Potential Melastatin 3 (TRPM3) ion channel has been suggested as a potential biomarker and target for therapeutics in people with ME/CFS, supported by a series of publications reporting genetic and protein changes. This study aimed to undertake a multi-site, large-scale investigation to determine the consistency of TRPM3 ion channel dysfunction in people with ME/CFS.

**Methods:**

TRPM3 ion channel activity was assessed in two distinct laboratory sites by independent investigators using whole-cell patch-clamp recordings performed in isolated natural killer (NK) cells from 36 ME/CFS participants, characterized according to the Canadian Consensus Criteria, and 42 healthy controls. The Mann–Whitney U test was used to compare endogenous TRPM3-like currents between cohorts. The effect of location was determined using a covariance analysis, while antagonist sensitivity was determined using Fisher’s Exact test.

**Results:**

Electrophysiological experiments revealed a significant reduction in TRPM3 function in NK cells from individuals diagnosed with ME/CFS compared with controls in all parameters analyzed. Importantly, there was no significant effect of the laboratory sites on the results of this investigation, which confirms TRPM3 as a consistent biomarker for ME/CFS.

**Conclusion:**

The current large-sample-size study confirmed previous results regarding TRPM3 ion channel dysfunction in NK cells in ME/CFS, demonstrating involvement of TRPM3 in the pathomechanism of this condition. Therefore, this multiple-site investigation offers strong evidence demonstrating TRPM3 as a potential biomarker for the diagnosis of ME/CFS, given the accumulating evidence.

## 1 Introduction

Myalgic Encephalomyelitis/Chronic Fatigue Syndrome (ME/CFS) is a serious condition characterized by the involvement of multiple organ systems, such as nervous, endocrine, immune, gastrointestinal, respiratory, and cardiovascular systems (1, 2). People with ME/CFS experience fatigue unrelieved by rest, post-exertional malaise (PEM), pain, dyspnea, orthostatic intolerance, cognitive disturbances, thermoregulatory dysfunction, and hypersensitive sense modalities (1–6). The ME/CFS symptomatology imposes significant disability levels and implications in individuals’ quality of life (QoL), which interferes with their participation in society (5, 7, 8).

ME/CFS aetiology remains elusive, and numerous factors have been postulated to explain this condition, such as genetic heritability, environmental causes, infections, stress, trauma, exposure to toxins, physical activity, and rest ratio (2, 9). Previous analysis has demonstrated that up to 75% of people with ME/CFS have experienced an infection-like episode preceding the onset of their condition; however, no single infectious agent is consistently associated with the development of ME/CFS (10–14). As of 2025, it is estimated that over 70 million people have ME/CFS globally; however, it is diagnosed based on clinical criteria, and the non-existence of a specific diagnostic test may result in an understated prevalence (1, 15–17). Hence, elucidating the pathomechanism and identifying biomarkers is crucial for advancing the development of diagnostic tests and enhancing access to healthcare and treatments.

The complex and multifactorial etiology of ME/CFS poses significant challenges to the development of an animal model to study the pathomechanisms of ME/CFS (5, 18, 19). In contrast, data obtained through multiple independent experiments and a meta-analysis demonstrated significant impairment in natural killer (NK) cell cytotoxic activity, which indicates that NK cells are a reproducible cell-based model to study ME/CFS (5, 13, 20–23). To date, alterations in NK cell quantity and cytotoxic activity are the most consistent immunological features in people with ME/CFS (20–22, 24–26). NK cells currently represent an accessible, validated, and relevant model to investigate ME/CFS (5, 13, 20–22).

Another notable advance in ME/CFS research was the identification of ME/CFS as a potential channelopathy, as demonstrated by a series of investigations that revealed the role of Transient Receptor Potential (TRP) ion channels in NK cells from affected individuals (27–33). In 2016, Marshall-Gradisnik et al. were the first researchers to associate differences in the TRP ion channel with NK cells from ME/CFS, reporting 11 single-nucleotide polymorphisms (SNPs) in TRP ion channel genes (*TRPC4*, *TRPC2*, *TRPM3,* and *TRPM8*) (27). In subsequent research, Transient Receptor Potential Melastatin 3 (TRPM3) cell surface expression was investigated and found to be significantly reduced on the surface of NK cells isolated from ME/CFS patients compared with HCs (34).

Fundamentally, the TRPM3 acts as a non-selective ion channel highly permeable to Ca^2+^ and contributes significantly to Ca^2+^ signaling regulation, which is critical for biological processes and intracellular pathways (30, 34–36). Notably, activation of TRPM3 results in a rise in intracellular Ca^2+,^ activating Ca^2+^-dependent intracellular signaling pathways, stimulating the recruitment of protein kinases, which play a role in cell differentiation and division, apoptosis, cell adhesion, immune synapse formation, degranulation, and release of cytolytic proteins, among others (30, 34–36). In contrast, dysregulation of TRPM3 function may affect intracellular Ca^2+^ concentration, which is negatively associated with impaired cell function and intracellular signaling pathways (29, 33, 37). Furthermore, patch-clamp experiments in NK cells from individuals with ME/CFS revealed significant reduction in TRPM3 ion channel activity compared to healthy controls (HC) after modulation with various agonists, as well as a significant increase in resistance to the TRPM3 antagonist agent (29, 37). These electrophysiology findings were reproduced by live-cell immunofluorescent imaging experiments, which confirmed a significant reduction in Ca^2+^ influx via TRPM3 in NK cells isolated from individuals with ME/CFS compared with HC (33).

Cumulatively, this emerging body of research suggests that the TRPM3 ion channel plays a crucial role in the etiology and pathomechanism of ME/CFS, given the cellular impairment and disruption of homeostasis. Validation of these findings in ME/CFS in a large cohort of patients and HC further supports the role of TRPM3 in the etiology and pathomechanism of ME/CFS. Therefore, the present investigation aimed to analyze the role of TRPM3 ion channel disturbances through a multisite collaborative investigation to evaluate the impact of TRPM3 impairment in the pathomechanism of this condition.

## 2 Materials and methods

### 2.1 Recruitment

For this study, 78 participants were recruited through the National Centre for Neuroimmunology and Emerging Diseases (NCNED) patient database and subdivided into two groups: 42 individuals in the HC group and 36 individuals in the ME/CFS group. All volunteers registered in the NCNED patient database completed a questionnaire that included questions regarding their demographic information, health history, medications, symptomatology (for ME/CFS patients), QoL, and disability.

The inclusion criteria for both groups included participants who resided in Australia, aged between 18 and 65 years, with a body mass index (BMI) ≤ 18 kg/m^2^ and ≥ 33.5 kg/m^2^. In addition, to be included in this study, volunteers must not report a history of chronic illness, such as autoimmune diseases, cardiovascular disease, diabetes, thyroid conditions, malignancies, or primary psychological disorders. Individuals reporting alcohol abuse, smoking, opioid use, being pregnant, breastfeeding, or lactating at the time of blood collection were excluded. Individuals taking medications or supplements that affect TRPM3 activity or Ca^2+^ signaling had the option to cease them temporarily when authorized by their physician and for a period determined based on the pharmacokinetics of medications or supplements. The washout period was determined for each compound based on pharmacokinetic parameters, such as the specific bioaccumulation characteristics and elimination half-life, to ensure sufficient time for clearance before blood donation. Participants who were unable or chose not to discontinue medication or supplements during the washout period requested during the recruitment phase were not included in this investigation before their blood donation. Moreover, eligibility for the HC group required self-reported absence of fatigue, showing no signs of illness, and being in good health, while individuals from the ME/CFS group had a confirmed medical diagnosis of ME/CFS and met the Canadian Consensus Criteria (CCC) or International Consensus Criteria (ICC) (1, 16).

This investigation was approved by the Griffith University Human Research Ethics Committee (GU HREC 2022/66). All participants received comprehensive information about their participation in this research and provided written consent before blood collection.

### 2.2 QoL, disability assessment, and symptoms

Participants were required to complete the NCNED Research Registry Questionnaire, which collects data on sociodemographic background, medical history, symptom presentation, and QoL across four sections. Symptom presentation was determined by recording the presence of a symptom within the last month, the frequency of the symptom (a little of the time, some of the time, a good bit of the time, most of the time, and all of the time), and the severity of the symptom (very mild, mild, moderate, severe, and very severe), aligning with the Centers for Disease Control and Prevention Symptom Inventory Questionnaire for CFS whilst incorporating additional symptoms to capture more recent diagnostic criteria. Symptoms were categorized as the presence or absence of fatigue, cognitive difficulties, pain, sleep disturbances, sensory disturbances, immune disturbances, gastrointestinal disturbances, cardiovascular symptoms, respiratory symptoms, thermostatic intolerances, and urinary disturbances. Burden of disability and QoL were determined using the World Health Organisation Disability Assessment Schedule (WHODAS) (38, 39) and the 36-item short-form health survey (SF-36) (40, 41), respectively. The WHODAS assesses functional capacity across six domains: communication and understanding, mobility, self-care, interpersonal relationships, life activities, and participation in society. The SF-36 comprises eight QoL domains: physical functioning, role limitations due to physical health problems, pain, general health, vitality, social functioning, role limitations due to personal or emotional problems, and emotional wellbeing. The score of WHODAS is inversely proportional to functional capacity (100% indicates full disability), while high scores in SF-36 indicate high QoL.

### 2.3 Blood collection and NK cells isolation

All participants donated samples of up to 84 ml of whole blood via venipuncture, collected in ethylenediaminetetraacetic acid (EDTA) tubes. Blood samples were collected at locations including Griffith University Gold Coast Campus, hospitals, or private laboratories in South-East Queensland, North-East New South Wales, and Perth (Western Australia). For the participants who could not travel to one of these places, a home visit was conducted for blood collection. After collection, 4 ml of blood samples were sent for full blood count (FBC) analysis in private laboratories. The remaining blood sample from each participant was sent to the NCNED at Griffith University on the Gold Coast campus or to the Ben Beale Laboratory in Cardiovascular Research at The University of Western Australia (UWA) for isolation of NK cells and performance of whole-cell patch-clamp experiments.

In the research facilities, Peripheral Blood Mononuclear Cells (PBMC) were isolated through centrifugation over a density gradient medium using Ficoll–Paque Premium (GE Healthcare, Uppsala, Sweden), as previously described (20, 42). Immediately after PBMC isolation, cells were adjusted to a final concentration of 5×10^7^ cells/ml to perform NK cell isolation by immunomagnetic negative selection using the EasySep Negative Human NK Cell Isolation (Stem Cell Technologies, Vancouver, BC, Canada). NK cell purity analysis was determined using the FortessaTM X-20 flow cytometer, AccuriC6 flow cytometer, or BD FACS Canto II (Becton Dickinson Biosciences (BD), San Diego, CA, USA). NK cells were incubated in the presence of CD56 APC (0.25 μg/20 μl) and CD3 PE Cy7 (0.25 μg/5 μl) monoclonal antibodies (BD Bioscience, San Jose, CA, USA), and the NK cell population was identified using phenotypic surface expression as CD3^−^CD56^+^.

### 2.4 Electrophysiological experiments

Manual whole-cell patch-clamp experiments were conducted in two research laboratories in Australia using the same intracellular pipette solution and extracellular solution, as described in previous small-sample studies that assessed TRPM3 in individuals with ME/CFS (29, 37). The glass pipettes were prepared using borosilicate glass capillaries (Harvard Apparatus, Holliston, MA, USA), and the pipette resistance varied from 8 to 12 MΩ. Patch-clamp recording data were filtered at 5 kHz and digitized at 10 kHz using Digidata 1440A (NCNED) and Digidata 1550B (UWA) analog-to-digital converter (Molecular Devices, Sunnyvale, CA, USA) and recorded using pClamp 10.7 software (Molecular Devices, Sunnyvale, CA, USA). Other equipment included in the experiment set-up are CV203BU head-stage (Molecular Devices, Sunnyvale, CA, USA), a three-way coarse manipulator, micro-manipulator (Narishige, Tokyo, Japan (NCNED) and from Sutter Instruments CA, USA (UWA)), and Axopatch 200B amplifier.

The voltage-ramp protocol was a holding potential of +10 mV to −90 mV, followed by a 0.1 s ramp to +110 mV, before returning to +10 mV (repeated every 10 s). The liquid junction potential between the glass pipette and extracellular solution (−10 mV) was corrected, but no subtraction of leak current components was undertaken. TRPM3 ion channel activity was activated by 100 μM Pregnenolone sulfate (PregS) (Tocris Bioscience, Bristol, UK) and suppressed by 10 μM ononetin (Tocris Bioscience, Bristol, UK), combined with 100 μM PregS, as previously described (29, 33, 43–45). Experiments were conducted at room temperature.

### 2.5 Data analysis

Sociodemographic, disability, FBC parameters, and purity were analyzed using the Statistical Package for the Social Sciences (SPSS) software, version 30 (IBM Corp, Armonk, NY, USA). NK cells are considered sensitive to ononetin when TRPM3 responses evoked by PregS (TRPM3 agonist) and were successfully inhibited by ononetin (TRPM3 antagonist) in the presence of PregS. A quality assurance analysis was performed on patch-clamp recordings to ensure the accuracy of electrophysiological data prior to statistical analysis. Following the analyses and interpretations conducted by the blind researcher who performed laboratory experiments, another blind researcher reviewed all data. Electrophysiological data were analyzed using pCLAMP 10.7 software (Molecular Devices, Sunnyvale, CA, USA), Origin 2021 (OriginLab Corporation, Northampton, MA, USA), GraphPad Prism version 10 (GraphPad Software Inc., La Jolla, CA, USA), and SPSS. Outliers were removed using the ROUT method, while normality of the distribution was analyzed via histogram plots and the Shapiro–Wilk normality test. Each patch-camp recording was considered a separate technical measurement during statistical analysis. The independent non-parametric Mann–Whitney U test was performed for statistical analysis of PregS and ononetin amplitudes, and Fisher’s exact test (Bonferroni method) was used to determine NK cells’ sensitivity to ononetin.

A covariance analysis (ANCOVA) was used to determine whether TRPM3 ion channel function differed between people with ME/CFS and HC when controlling for laboratories. Given the inclusion of samples processed in two different laboratories, each with differences in setup and experimenters, the effect of different laboratories on this research outcome was analyzed.

## 3 Results

### 3.1 Participant characteristics and blood parameters

The demographic parameters and FBC results from all 78 participants are summarized in Table 1. No statistical significance was identified in age, gender, BMI or level of education achieved between the ME/CFS and HC groups. The participation of women was higher in both groups in this study (66.7% in HC and 61.1% in ME/CFS), consistent with previous research. The average age was 44.21 ± 11.44 and 44.19 ± 11.63 in HC and ME/CFS cohorts, respectively. In contrast, the employment status was significantly different between ME/CFS and HC (p < 0.001), with 63.4% of HC reporting full-time employment while only 13.9% of ME/CFS patients did. Employment status was the unique demographic parameter that differed between groups. This difference is expected given the impact of the debilitating symptoms experienced by ME/CFS patients. This is reflected by 25% of ME/CFS participants reporting illness/disability as their employment status, contrasting with no HC reporting illness/disability. Regarding the FBC results, ME/CFS participants reported a significant reduction in the total white cell count (*p* = 0.005) and neutrophils (*p* = 0.010) compared to the HC.

**Table 1:** Participant demographics and FBC parameters.

Demographics	FBC	HC	ME/CFS	*p*-value
Age (years)		44.21 ± 11.44	44.19 ± 11.63	0.916
Gender *N* (%)	Male	14 (33.3%)	14 (38.9%)	0.612
	Female	28 (66.7%)	22 (61.1%)	
BMI (kg/m^2^)		24.57 ± 3.48	23.65 ± 3.47	0.219
Employment status	Full time	26 (63.4%)	5 (13.9%)	**<0.001**
	Part time	7 (17.1%)	5 (13.9%)	
	Casual	5 (12.2%)	3 (8.3%)	
	Unemployed	3 (7.3%)	14 (38.9%)	
	Illness/disability	0 (0.0%)	9 (25.0%)	
Education	Primary education	0 (0.0%)	0 (0.0%)	0.987
	High school	6 (14.6%)	6 (16.7%)	
	Undergraduate	12 (29.3%)	13 (36.1%)	
	Postgraduate/doctoral	18 (43.9%)	8 (22.2%)	
	Other	5 (12.2%)	9 (25.0%)	
Full blood count	White cell count (4.0–11.0 ×10^9^/L)	6.01 ± 0.95	5.42 ± 1.22	**0.005**
	Lymphocytes (1.0–4.0 ×10^9^/L)	1.94 ± 0.67	1.84 ± 0.63	0.486
	Neutrophils (2.0–8.0 ×10^9^/L)	3.40 ± 0.80	2.98 ± 0.97	**0.010**
	Monocytes (0.1–1.0 ×10^9^/L)	0.47 ± 0.13	0.42 ± 0.10	0.078
	Eosinophils (< 0.6 ×10^9^/L)	0.17 ± 0.10	0.15 ± 0.11	0.303
	Basophils (< 0.2 ×10^9^/L)	0.05 ± 0.02	0.05 ± 0.03	0.827
	Platelets (140–400 ×10^9^/L)	256.2 ± 49.05	247.7 ± 43.73	0.499
	Red cell count (3.8–5.2 ×10^12^/L)	4.66 ± 0.65	4.67 ± 0.50	0.528
	Hematocrit (0.33–0.47)	0.41 ± 0.04	0.42 ± 0.04	0.435
	Hemoglobin (115–160 g/L)	137.1 ± 13.68	140.5 ± 13.79	0.279

Data are presented as mean ± SD or *N* (%). Missing data from the HC participant who did not complete the education and employment status. Values of *p* < 0.05 are bolded. Reference ranges for full blood count parameters have been included in the table. BMI, body mass index; FBC, full blood count; HC, healthy controls; ME/CFS, myalgic encephalomyelitis/chronic fatigue syndrome.

Data from this investigation identified significant differences between cohorts in all SF-36 and WHODAS domains analyzed. As detailed in Table 2, all eight SF-36 domains were significantly reduced (<0.001) in the ME/CFS cohort compared with HC. Additionally, all six WHODAS domains were significantly increased in ME/CFS compared with HC (<0.001). As expected, and in line with previous research, the patients with ME/CFS in this study had a significantly decreased QoL and significantly increased disability level (<0.001).

**Table 2:** Participant Quality of Life and Disability Scores.

SF-36 (%)	HC	ME/CFS	*p*-value
Physical functioning	96.19 ± 13.01	44.71 ± 29.41	**<0.001**
Physical role	96.58 ± 15.87	19.86 ± 18.23	**<0.001**
Pain	92.92 ± 12.60	51.40 ± 27.03	**<0.001**
General health	77.88 ± 11.29	31.13 ± 16.71	**<0.001**
Social functioning	93.45 ± 13.31	30.88 ± 25.79	**<0.001**
Emotional role	93.85 ± 10.59	66.67 ± 29.23	**<0.001**
Emotional wellbeing	82.57 ± 14.80	58.97 ± 21.59	**<0.001**
Vitality	71.73 ± 18.33	16.55 ± 16.56	**<0.001**
WHODAS (%)			
Communication and understanding	5.85 ± 10.49	44.64 ± 20.50	**<0.001**
Mobility	1.07 ± 3.58	43.14 ± 25.58	**<0.001**
Self-care	0.30 ± 1.93	22.51 ± 25.51	**<0.001**
Interpersonal relationships	3.57 ± 7.45	36.61 ± 26.00	**<0.001**
Life activities	1.94 ± 5.60	57.68 ± 28.04	**<0.001**
Participation in society	3.87 ± 9.28	53.67 ± 24.73	**<0.001**

Data are presented as mean ± SD. The WHODAS domain participation in life activities involving work or school is not included due to high unemployment rates in people with ME/CFS. Values of *p* < 0.05 are bold. HC, healthy controls; ME/CFS, myalgic encephalomyelitis/chronic fatigue syndrome; SF-36, 36-item short-form health survey; WHODAS, World Health Organization disability assessment schedule.

The symptomatology and disease history from the ME/CFS cohort are presented in Table 3. The ME/CFS population is characterized by an average age of onset of 30.63 ± 13.31 years and a disease duration of 13.40 ± 8.36 years. An infectious insult was the predominant reported onset by ME/CFS individuals (80.0%, *N* = 24), while the other ME/CFS participants reported stress (10%, *N* = 03), vaccine (3.33%, *N* = 1), and life events (6.67%, *N* = 2). Six ME/CFS patients provided no information and thus were not considered for the onset assessment. Fatigue, pain, and sleep disturbances were reported by all ME/CFS participants. Other frequently observed symptoms in ME/CFS participants included cognitive difficulties (97.22%, *N* = 35), sensory disturbances (88.89%, *N* = 32), and immune disturbances (80.56%, *N* = 29).

**Table 3:** Symptoms and onset details from ME/CFS group.

Variables	Symptoms	ME/CFS
Age of onset (Years [Mean ± SD])		30.63 ± 13.31
Disease duration (Years [Mean ± SD])		13.40 ± 8.36
Infectious onset, *N* (%)		24 (80.0%)
Fatigue	Yes	36 (100.0%)
	No	0 (0.0%)
Cognitive difficulties	Yes	35 (97.22%)
	No	1 (2.78%)
Pain	Yes	36 (100.0%)
	No	0 (0.0%)
Sleep disturbances	Yes	36 (100.0%)
	No	0 (0.0%)
Sensory disturbances	Yes	32 (88.89%)
	No	4 (11.11%)
Immune disturbances	Yes	29 (80.56%)
	No	7 (19.44%)
Gastrointestinal disturbances	Yes	25 (73.53%)
	No	9 (26.47%)
Cardiovascular symptoms	Yes	25 (69.44%)
	No	11 (30.56%)
Respiratory symptoms	Yes	21 (58.33%)
	No	15 (41.67%)
Thermostatic intolerance	Yes	26 (74.29%)
	No	9 (25.71%)
Urinary disturbances	Yes	13 (36.11%)
	No	23 (63.89%)

Data presented as mean ± SD and *N* (%). Missing data regarding onset (*N* = 6), gastrointestinal symptoms (*N* = 2), and thermostatic instability symptoms (*N* = 1). HC, healthy control; ME/CFS, myalgic encephalomyelitis/chronic fatigue syndrome; N, number of participants.

In this multiple-site investigation, the demographic characteristics, symptomatology, and onset between ME/CFS, analyzed at the NCNED, Griffith University, and UWA were compared to assess the consistency of the ME/CFS sample population. Interestingly, the only significant difference identified in the parameters analyzed was regarding the cardiovascular symptoms, which were reported by 80.77% and 40.0% in Griffith and UWA samples, respectively (Table 4).

**Table 4:** ME/CFS demographic, symptoms, and onset details compared by location.

Variables		ME/CFS Griffith	ME/CFS UWA	*p*-value
Age (years)		42.23 ± 10.91	49.30 ± 12.45	0.097
Gender N (%)	Male	9 (34.62%)	5 (50.0%)	0.403
	Female	17 (65.38%)	5 (50.0%)	
BMI (kg/m^2^)		23.25 ± 3.62	24.69 ± 2.96	0.163
Employment status	Full time	3 (11.5%)	2 (20.0%)	0.211
	Part time	4 (15.4%)	1 (10.0%)	
	Casual	1 (3.8%)	2 (20.0%)	
	Unemployed	9 (34.6%)	5 (50.0%)	
	Illness/disability	9 (34.6%)	0 (0.0%)	
Education	Primary education	0 (0.0%)	0 (0.0%)	0.101
	High school	4 (15.4%)	2 (20.0%)	
	Undergraduate	8 (30.8%)	5 (50.0%)	
	Postgraduate/doctoral	6 (23.1%)	2 (20.0%)	
	Other	8 (30.8%)	1 (10.0%)	
Onset Details	Age of onset (Years [Mean ± SD])	29.36 ± 12.25	33.80 ± 15.92	0.381
	Disease duration (Years [Mean ± SD])	12.56 ± 8.30	15.50 ± 8.58	0.333
	Infectious onset, N (%)	16 (76.19%)	8 (88.89%)	0.442
Symptoms	Fatigue	26 (100%)	10 (100%)	>0.999
	Cognitive difficulties	25 (96.15%)	10 (100.0%)	0.535
	Pain	26 (100%)	10 (100%)	>0.999
	Sleep disturbances	26 (100%)	10 (100%)	>0.999
	Sensory disturbances	24 (92.31%)	8 (80.0%)	0.299
	Immune disturbances	21 (80.77%)	8 (80.0%)	0.959
	Gastrointestinal disturbances	16 (66.67%)	9 (90.0%)	0.166
	Cardiovascular symptoms	21 (80.77%)	4 (40.0%)	**0.019**
	Respiratory symptoms	16 (61.54%)	5 (50.0%)	0.535
	Thermostatic intolerance	18 (72.0%)	8 (80.0%)	0.630
	Urinary disturbances	10 (38.46%)	3 (30.0%)	0.641

Data presented as mean ± SD or *N* (%). Missing data regarding onset (*N* = 6), gastrointestinal symptoms (*N* = 2), and thermostatic instability symptoms (*N* = 1). Values of *p* < 0.05 are bold. BMI, body mass index; ME/CFS, myalgic encephalomyelitis/chronic fatigue syndrome; N, number of participants; QLD, Queensland; WA, Western Australia.

### 3.2 Electrophysiological experiments

The whole-cell patch-clamp technique was performed to assess TRPM3 ion channel function in NK cells from ME/CFS and HC individuals. In this investigation, flow cytometry was used to evaluate the percentage of NK cells obtained through negative selection, ensuring that only samples with a high purity of NK cells (>85%) were included in electrophysiological experiments. NK cell purity was 92.92% ± 4.71 for HC and 92.86% ± 3.52 for ME/CFS patients. No statistical difference was observed between cohorts regarding NK cell purity (*p* = 0.5413). The statistical analysis includes 552 whole-cell patch-clamp recordings. Regarding PregS stimulation, 248 recordings from NK cells from ME/CFS and 304 from HC were taken. Given that the viability of NK cells during perfusion protocols with pharmacological agents or current was unstable during the second half of recording, the number of ononetin analyses included 452 recordings from 195 ME/CFS and 257 HC NK cells. NCNED processed 33 HC and 26 ME/CFS samples, while UWA processed 9 samples from HC and 10 from ME/CFS patients.

**Figure 1:**
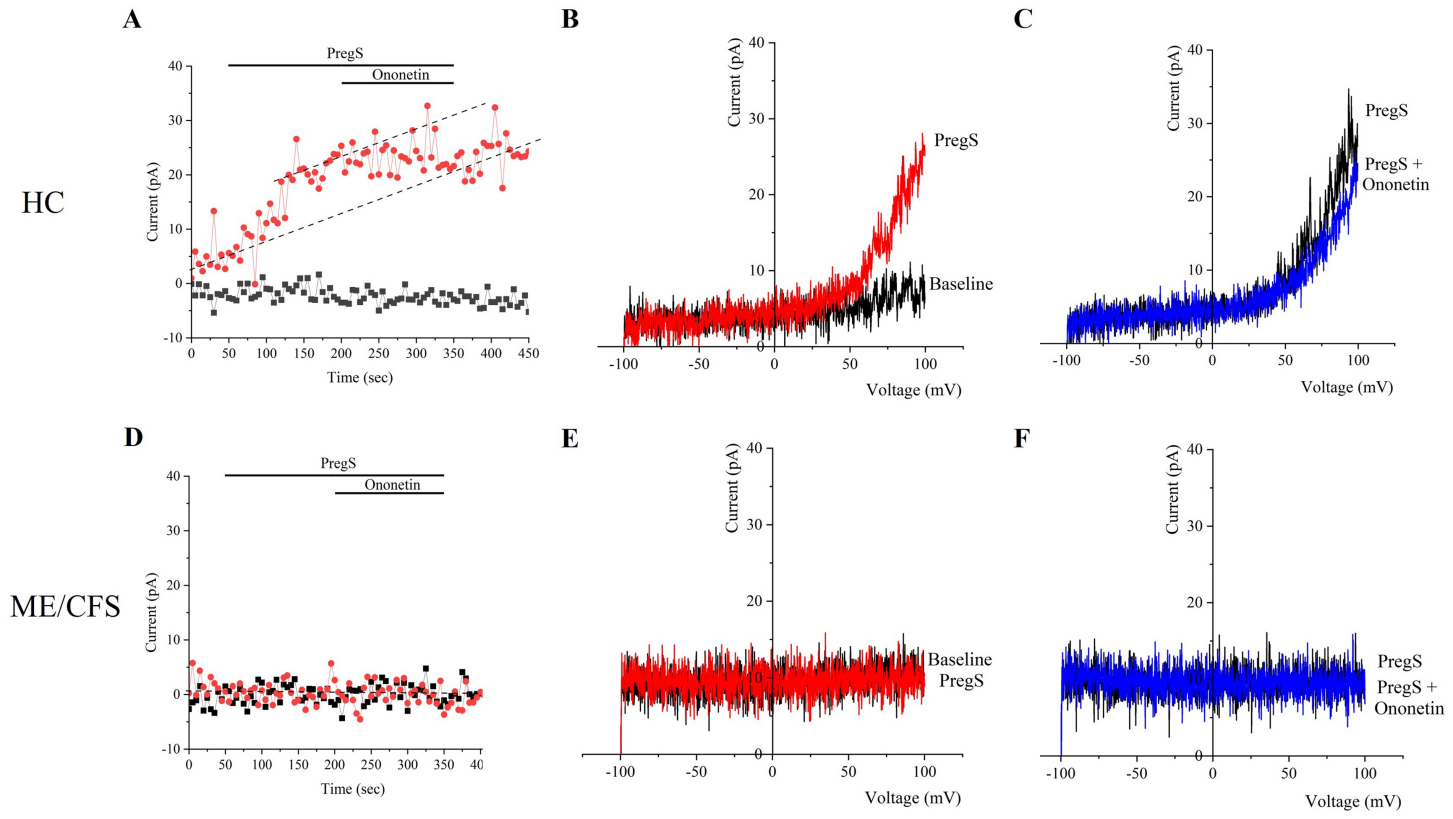
TRPM3 ion channel activity in NK cells from HC and ME/CFS. **(A–C)** Example of a recording on an NK cell from HC. **(A)** A representative time-series of a current at +100 mV and −100 mV. **(B)** Representative I–V curve showing results before and at the end of PregS stimulation. **(C)**. Representative I–V curve measured at the conclusion of PregS stimulation and following ononetin exposure. **(D–F)** Example of a recording on an NK cell from a person diagnosed with ME/CFS. **D**. A representative time-series of a current amplitude at +100 mV and −100 mV. **(E)** Representative I–V curve showing results before and at the end of PregS stimulation. **(F)** Representative I–V curve measured at the conclusion of PregS stimulation and following ononetin exposure. HC, healthy control; ME/CFS, Myalgic Encephalomyelitis/Chronic Fatigue Syndrome; NK, natural killer; PregS, pregnenolone sulfate.

**Figure 2:**
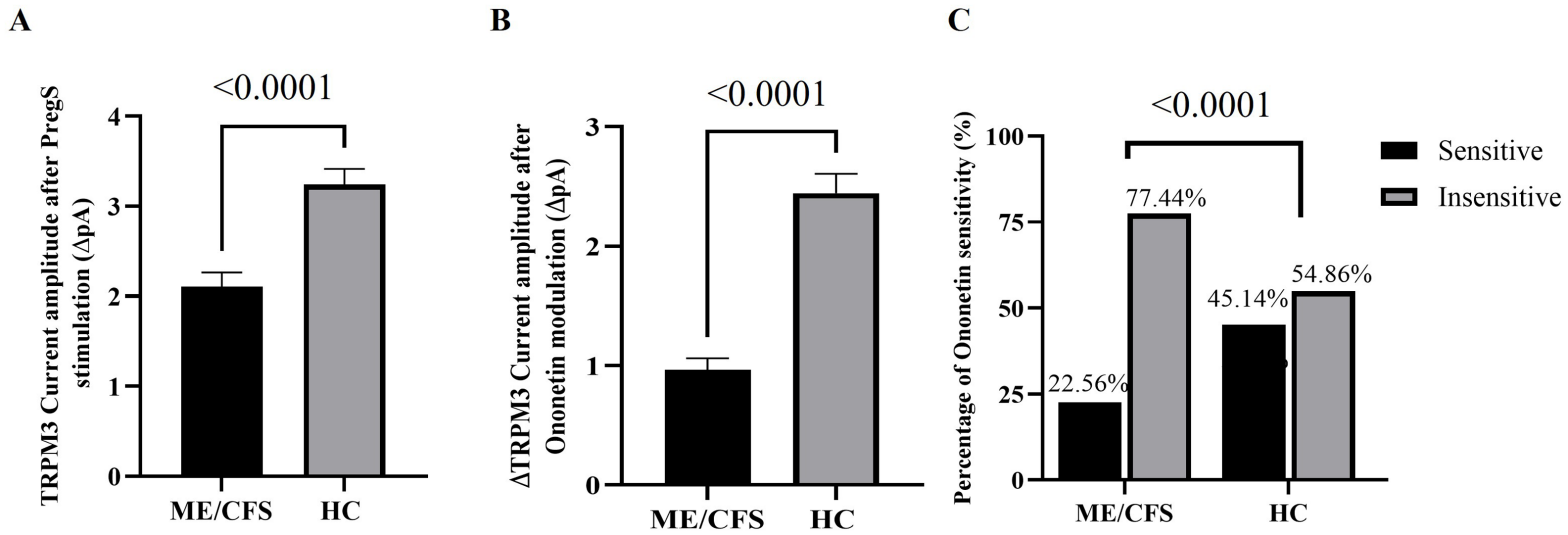
Statistical analysis of TRPM3 ion channel activity in ME/CFS and HC cohorts. **(A)** Bar graph representing TRPM3 current amplitude at +100 mV following PregS stimulation in ME/CFS (*N* = 36; *n* = 248) and HC (*N* = 42; *n* = 304). **(B)** Bar graphs representing TRPM3 inhibition after application of ononetin in ME/CFS (*N* = 36; *n* = 195) and HC (*N* = 42; *n* = 257). **(C)** Bar graphs illustrate the percentage of NK cells sensitive and insensitive to ononetin response. N indicates the number of participants, while n shows the number of records included. Data are presented as mean ± SEM, and Mann–Whitney U test for PregS/ononetin amplitudes, and Fisher’s exact test (Bonferroni method) are applied for analyzing the number of NK cells sensitive to ononetin comparing the cohorts. HC, healthy control; Myalgic Encephalomyelitis/Chronic Fatigue Syndrome; NK, natural killer; PregS, pregnenolone sulfate.

PregS (100 μM) was the agonist used to stimulate TRPM3 ion channel currents, and ononetin (10 μM) was the antagonist agent applied in combination with PregS to inhibit PregS-evoked currents and confirm the involvement of TRPM3 ion channels. Voltage-clamp recordings revealed that the predominant number of NK cells from the HC cohort had small outward rectifying currents induced by application of PregS and a voltage relationship (I–V) curve typical of TRPM3 ion channels, as illustrated in Figures 1A,B. The results observed in NK cells from HC are characteristic of TRPM3 ion channels, as stimulation with PregS increases the movement of Ca^2+^ through TRPM3, potentially leading to an increase in intracellular Ca^2+^ concentration (Figure 2A). In contrast to the findings in the HC cohort, only a few NK cells from ME/CFS patients exhibited an increase in currents upon stimulation with PregS, demonstrating that most NK cells from ME/CFS have impaired TRPM3 function (Figure 2A), as represented in Figure 1D,E. In the second part of the recordings, ononetin was added to the perfusion solution in combination with PregS to suppress TRPM3 currents evoked by PregS stimulation. Ononetin effects on TRPM3 ion channel function confirmed results obtained with PregS. NK cells from HC exhibited PregS-evoked currents that were inhibited by ononetin application, and an I-V curve response was observed as expected in a cell with a functional TRPM3 ion channel (Figure 1C). In contrast to the results from NK cells of HC participants, the ME/CFS cohort had a reduced or no response upon modulation with ononetin (Figure 1F) as only 22.56% (number of recordings = 44) of NK cells from ME/CFS were sensitive to ononetin, in comparison to 77.44% (number of recordings = 151) of NK cells from HC. These findings confirmed the results obtained with PregS, indicating that TRPM3 channels are dysfunctional in NK cells isolated from ME/CFS patients.

To evaluate the function of the TRPM3 ion channel through statistical analysis, amplitudes were calculated for individual recordings. PregS amplitude was determined as the difference between the baseline level and the end of PregS application, while ononetin amplitude was quantified as the difference between the termination of PregS stimulation and the end of ononetin application, both illustrated in the time-series graphs in Figures 1A,D. A significant reduction in PregS-evoked ionic currents, and amplitude of ononetin was observed in NK cells from people with ME/CFS when compared to NK cells from HC individuals (both *p* < 0.0001), confirming findings reported in small sample studies (29, 32, 37). ANCOVA analysis confirmed that different research facilities were not a confounding factor in this study. The laboratory’s effect was not statistically significant for PregS amplitude (*p* = 0.918) and ononetin amplitude (*p* = 0.369), demonstrating that TRPM3 ion channel dysfunction in ME/CFS patients was reproducible across two distinct laboratories, using different experimenters, with a variance of 0.001% for PregS and 0.02% for ononetin. In addition, the number of NK cells sensitive to ononetin was also significantly reduced in cells from ME/CFS compared to those from HC (*p* < 0.0001). Figure 2 provides further details on the statistical results.

## 4 Discussion

The identification of TRPM3 ion channel proteins on the surface of NK cells from HC provided evidence of TRPM3 expression, and a reduction in TRPM3 surface expression in ME/CFS suggested potential implications of this ion channel function in disease (34). TRPM3 expression on the surface of NK cells was quantified using specific antibodies through flow cytometry and further complemented by identification of TRPM3 in NK cells using liquid chromatography–multiple reaction monitoring (LC-MRM) (46). Given that our previous research has identified TRPM3 ion channel expression on the surface of NK cells and the importance of Ca^2+^ sustained mobilization for NK cell function, TRPM3 dysfunction may contribute to impaired NK cell activity in ME/CFS (28, 33, 34).

The current study is the first large-scale research to analyze endogenous TRPM3 ion channel function in people diagnosed with ME/CFS, contributing to the elucidation of the pathomechanism of this debilitating condition. The findings of our previous single-laboratory investigations are confirmed by the present study, which incorporates electrophysiological analysis conducted in two independent laboratories in patients from different Australian regions. This multi-center approach, combined with a large sample size investigation, permitted the assessment of the reproducibility of TRPM3 function as a robust biomarker for ME/CFS.

Using the gold standard technique for research on ion channels, this study demonstrated that peripheral NK cells from ME patients exhibited impaired TRPM3 response compared to NK cells isolated from HC participants. We identified a significant reduction in TRPM3 ion channel function in NK cells isolated from ME/CFS patients, both in response to stimulation with the agonist PregS (*p* < 0.0001) and in suppression of activation using ononetin (*p* < 0.0001). The current results are consistent with those of Cabanas et al. and Eaton-Fitch et al., who investigated TRPM3 by patch-clamp and the impact of TRPM3 dysfunction on Ca^2+^ mobilization through live-cell imaging in NK cells from people with ME/CFS, respectively (29, 33, 37).

Impairment in TRPM3 ion channels in people with ME/CFS leads to disruption in Ca^2+^ influx, which causes disruptions in cellular activity through alterations in intracellular signaling cascades (28, 47, 48). Two mechanisms promote Ca^2+^ influx through TRPM3 channels: the depletion of Ca^2+^ intracellular stores and the activation of Gq-coupled muscarinic receptors; therefore, this suggests Ca^2+^-dependent activation of TRPM3 (48, 49). Furthermore, TRPM3 impairment adversely impacts cellular functions by reducing intracellular Ca^2+^ levels in various organs and tissues, with the consequences depending on the specific roles of affected cells (29, 33, 37).

Regarding immunological activities, Ca^2+^ plays a critical role in immunity activation, target cell adhesion, formation of the immunological synapse, regulation of antigen receptors and target cell recognition, secretion of cytolytic proteins, and NK cell cytotoxicity (30, 34–36, 48, 50–57). Immune disturbances were a key observation in the current study, with 80.56% of participants in the ME/CFS cohort reporting these symptoms. Current data, along with findings of reduced cytotoxic activity in NK cells from ME/CFS patients (21, 23, 58), suggest that impaired TRPM3 ion channel function may contribute to the NK cell dysfunction in people with ME/CFS.

As stated in the literature, TRPM3 is also widely expressed in human tissues and cells, including the central nervous system (CNS), muscle, liver, kidneys, pancreas, and cardiovascular organs (59, 60). Crucially, the abundant expression of TRPM3 is aligned with symptoms experienced by people diagnosed with ME/CFS (5). For instance, several neurological processes that demand Ca^2+^, including long-term cell survival, learning, and memory, are impacted by disturbances in Ca^2+^ homeostasis in the CNS (61, 62). Another neurological association of ME/CFS is related to the increase of glutamate in ME/CFS patients (63), which is particularly interesting given that TRPM3 participates in the control of spontaneous glutamate release in neurons (63, 64). Normal levels of glutamate contribute to memory, mood regulation, neurocognition, and neuroplasticity; however, increases lead to nerve cell death (65, 66). Remarkably, in the ME/CFS cohort of this investigation, cognitive difficulties were reported for all patients except one (97.22%), and sensory disturbances for 32 of 36 participants (88.89%), which is potentially linked to dysregulation of TRPM3 in the CNS of people with ME/CFS given the expression of TRPM3 in the CNS. It is postulated that a reduction in TRPM3 activity in the CNS leads to a significant decrease of Ca^2+^ intracellular concentration, affecting cellular function and causing neurological symptoms.

TRPM3 ion channels are expressed in nociceptive sensory neurons (in dorsal root ganglion and trigeminal ganglia), while TRPM3 activation leads to a release of calcitonin gene-related peptide (CGRP) from sensory nerve terminals, which is a process dependent on TRPM3 (45, 67, 68). Research also demonstrated TRPM3 function in heat hypersensitivity and spontaneous pain following nerve injury, but TRPM3 modulation was without effect in cold or mechanical hyperalgesia (69). Mulier et al. reported increased *Trpm3* mRNA levels in dorsal root ganglion neurons innervating mice’s inflamed paws and increased TRP-mediated Ca^2+^ response, demonstrating that TRPM3 plays a substantial role in the inflammatory hyperalgesia (70). Another mechanism potentially involved in this process is related to TRPM3 expression and function in vascular smooth muscle proliferation and contraction, leading to vasoconstriction (71, 72).

Pathways involved in the activation of G-protein-coupled receptors (GPCR) are associated with TRPM3 ion channel inhibition by the direct binding of Gβγ proteins (73–76). Interestingly, the broadly distributed opioid receptors belong to the GPCR family, and it is notable that they are activated by opioid medications, which are currently among the most potent analgesics (77). When activated, opioid receptors bind to heterotrimeric Gi/o proteins, leading to dissociation into Gαi/o and Gβγ subunits to modulate several signaling pathways (78). Notably, Gβγ subunits exert modulator effects in some ion channels, including inhibition of TRPM3 channels, which has been demonstrated by local activation of peripheral opioid receptors involving Gβγ proteins, leading to strong analgesia of TRPM3-dependent pain (77, 79, 80). This evidence supports the hypothesis of using opioid receptor antagonists, such as Naltrexone (NTX), to inhibit opioid receptors, which leads to alleviating TRPM3 suppression by Gβγ subunit (77, 81–85). *In vitro* studies on NK cells treated overnight with NTX confirmed the restoration of impaired TRPM3 ion channels in ME/CFS and long COVID patients (81, 86).

Following the COVID-19 pandemic, multiple studies have indicated that Severe Acute Respiratory Syndrome Coronavirus 2 (SARS-CoV-2) is a potential infectious trigger for ME/CFS (11–13, 87), given the elevation of new ME/CFS cases since the pandemic. This is further supported by abundant evidence that other viruses are correlated to the onset of ME/CFS, such as Epstein–Barr Virus, influenza, cytomegalovirus, Ross River virus, Q fever, enteroviruses, and other pathogens (11–14, 87). This is also demonstrated by the ME/CFS cohort in this study, which includes 80% of patients reporting an infectious onset for ME/CFS, in accordance with the literature, which reports that approximately three-quarters of ME/CFS cases have an onset following infection episodes (10). Interestingly, our previous investigations have reported a consistent overlap in TRPM3 dysfunction between patients with ME/CFS and individuals diagnosed with long COVID in recent publications (32, 86). Although the current findings from this study cannot be directly extrapolated to long COVID patients, it highlights the importance of further studies with a large cohort of long COVID patients to validate the investigations.

Previous research indicates the role of TRPM2, TRPM3, and TRPM7 ion channels in the pathomechanism of ME/CFS (29–31, 33, 34, 37, 46, 88, 89). However, further investigations into other TRP ion channels might determine their involvement and contribute to elucidating the ME/CFS pathomechanism. Findings from this investigation regarding TRPM3 function in individuals with ME/CFS provide further insight into ME/CFS pathomechanisms and are pivotal for advancing not only the development of a diagnostic test but also the identification and treatment based on the restoration of TRPM3 ion channel activity that relieves symptoms and improves QoL. TRPM3 is a current target for therapeutic interventions, as previously successfully demonstrated *in vitro* and in NK cells from ME patients on treatment with low-dose naltrexone to restore TRPM3 function and improve symptomatology (81, 82). Future double-blinded clinical trials may investigate whether the improvement in ME/CFS clinical presentation during low-dose naltrexone treatment is correlated with the restoration of TRPM3 function, Ca^2+^ re-establishment, and improved clinical manifestations.

## 5 Conclusion

Overall, findings from this inter-laboratory study provided crucial data to support TRPM3 as a potential biomarker for research and diagnosis of ME/CFS, as the first large sample size study investigating TRPM3 disruption in people diagnosed with ME/CFS compared to HC. Notably, the symptomatic presentation of ME/CFS is associated with the distribution of the TRPM3 ion channels, as demonstrated by previous investigations into the effects of TRPM3 dysregulation and a consequential reduction in intracellular Ca^2+^ in various tissues and organs, investigated in both human and animal models.

## Data Availability

The datasets presented in this article are not readily available because the original contributions presented in the study are included in the article/supplementary material, further inquiries can be directed to the corresponding author/s. Requests to access the datasets should be directed to ncned@griffith.edu.au.
